# Using the Progression Independent of Relapse Activity Framework to Unveil the Pathobiological Foundations of Multiple Sclerosis

**DOI:** 10.1212/WNL.0000000000209444

**Published:** 2024-06-18

**Authors:** Olga Ciccarelli, Frederik Barkhof, Massimiliano Calabrese, Nicola De Stefano, Arman Eshaghi, Massimo Filippi, Claudio Gasperini, Cristina Granziera, Ludwig Kappos, Maria A. Rocca, Àlex Rovira, Jaume Sastre-Garriga, Maria Pia Sormani, Carmen Tur, Ahmed T. Toosy, as the MAGNIMS Study Group

**Affiliations:** From the Queen Square MS Centre (O.C., F.B., A.E., A.T.T.), Department of Neuroinflammation, UCL Queen Square Institute of Neurology, Faculty of Brain Sciences, University College London; National Institute for Health and Care Research (NIHR) (O.C.), University College London Hospitals (UCLH) Biomedical Research Centre; Centre for Medical Image Computing (F.B.), University College London, United Kingdom; Department of Radiology and Nuclear Medicine (F.B.), Amsterdam UMC, Vrije Universiteit Amsterdam, the Netherlands; Department of Neurosciences, Biomedicine and Movement Sciences (M.C.), University of Verona; Department of Medicine, Surgery and Neuroscience (N.D.S.), University of Siena; Neuroimaging Research Unit (M.F., M.A.R.), Division of Neuroscience, and Neurology Unit (M.F., M.A.R.), Neurorehabilitation Unit, Neurophysiology Service, IRCCS San Raffaele Scientific Institute; Vita-Salute San Raffaele University (M.F., M.A.R.), Milan; Department of Neuroscience (C. Gasperini), San Camillo Hospital, Rome, Italy; Translational Imaging in Neurology (ThINK) Basel (C. Granziera, L.K.), Department of Biomedical Engineering, Faculty of Medicine, University Hospital Basel and University of Basel; Research Center for Clinical Neuroimmunology and Neuroscience Basel (RC2NB) (C. Granziera, L.K.); University Hospital Basel and University of Basel (C. Granziera, L.K.), Switzerland; Section of Neuroradiology (À.R.), Department of Radiology, and Multiple Sclerosis Centre of Catalonia (J.S.-G., C.T.), Department of Neurology, Hospital Universitari Vall d'Hebron, Universitat Autònoma de Barcelona, Spain; Department of Health Sciences (M.P.S.), University of Genova; and IRCCS Ospedale Policlinico San Martino (M.P.S.), Genova, Italy.

## Abstract

Progression independent of relapse activity (PIRA), a recent concept to formalize disability accrual in multiple sclerosis (MS) independent of relapses, has gained popularity as a potential clinical trial outcome. We discuss its shortcomings and appraise the challenges of implementing it in clinical settings, experimental trials, and research. The current definition of PIRA assumes that acute inflammation, which can manifest as a relapse, and neurodegeneration, manifesting as progressive disability accrual, can be disentangled by introducing specific time windows between the onset of relapses and the observed increase in disability. The term PIRMA (progression independent of relapse and MRI activity) was recently introduced to indicate disability accrual in the absence of both clinical relapses and new brain and spinal cord MRI lesions. Assessing PIRMA in clinical practice is highly challenging because it necessitates frequent clinical assessments and brain and spinal cord MRI scans. PIRA is commonly assessed using Expanded Disability Status Scale, a scale heavily weighted toward motor disability, whereas a more granular assessment of disability deterioration, including cognitive decline, using composite measures or other tools, such as digital tools, would possess greater utility. Similarly, using PIRA as an outcome measure in randomized clinical trials is also challenging and requires methodological considerations. The underpinning pathobiology of disability accumulation, that is not associated with relapses, may encompass chronic active lesions (slowly expanding lesions and paramagnetic rim lesions), cortical lesions, brain and spinal cord atrophy, particularly in the gray matter, diffuse and focal microglial activation, persistent leptomeningeal enhancement, and white matter tract damage. We propose to use PIRA to understand the main determinant of disability accrual in observational, cohort studies, where regular MRI scans are not included, and introduce the term of “advanced-PIRMA” to investigate the contributions to disability accrual of the abovementioned processes, using conventional and advanced imaging. This is supported by the knowledge that MRI reflects the MS pathogenic mechanisms better than purely clinical descriptors. Any residual disability accrual, which remains unexplained after considering all these mechanisms with imaging, will highlight future research priorities to help complete our understanding of MS pathogenesis.

## Introduction

**Figure 1:**
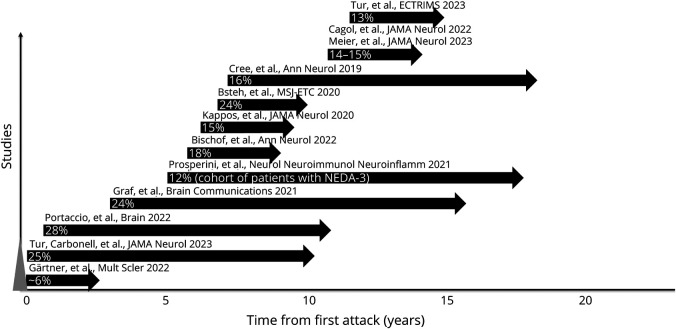
Annual Frequency of PIRA Events Reported by Previous Studies This figure represents the percentage of people with MS who develop at least 1 PIRA event a year, as reported by previous studies. Each study is represented by an arrow, whose length indicates the median time of the study follow-up. The position of the start of the arrow along the x-axis represents the median/mean (as available) disease duration of the patients at study entry. The references are given in the eTable 1. MS = multiple sclerosis; NEDA = no evidence of disease activity; PIRA = progression independent of relapse activity.

Conventionally, disability accrual in relapsing remitting multiple sclerosis (RRMS) is thought to occur because of (1) incomplete recovery after a relapse (relapse-associated worsening [RAW]) and (2) progression independent of relapse activity (PIRA).^1^ PIRA is a clinical concept that represents insidious disability accrual not influenced by relapses, including preceding, concurrent, and succeeding relapses^2^ (the term disability accrual is used to describe any observed increase in disability within the context of PIRA, irrespective of the underlying phenotype). PIRA brings the concept of disability accrual independent of relapses, considered to be exclusive to patients with progressive MS,^3^ to the RRMS phenotype, even in early MS^4^ and after the first demyelinating event.^5^ PIRA appears to be the main driver of disability accumulation across all MS phenotypes.^1,6-8^ We estimate that the proportion of patients developing PIRA is 3%–4% per each year of follow-up (Figure 1). The underlying mechanisms of PIRA are thought to be chronic inflammation and neurodegeneration, more pronounced in progressive MS, but also present in relapsing MS.^9^

Herein, we discuss the challenges facing PIRA, from its clinical-based definition to its translation into clinical settings and trials, and the likely biological underpinning PIRA, as assessed by imaging. In response to calls to classify MS rooted in its biological mechanisms,^10^ we propose to evolve from a clinical-based definition of PIRA toward a comprehensive quantification of pathogenic mechanisms related to PIRA using MRI and other paraclinical measures of underlying pathology. This commentary arises from the themes discussed at a Magnetic Resonance Imaging in MS (MAGNIMS) consortium workshop dedicated to PIRA, held in May 2023 in Verona, Italy.

## Shortcomings of PIRA Definition in Light of MS Pathogenic Mechanisms

**Figure 2:**
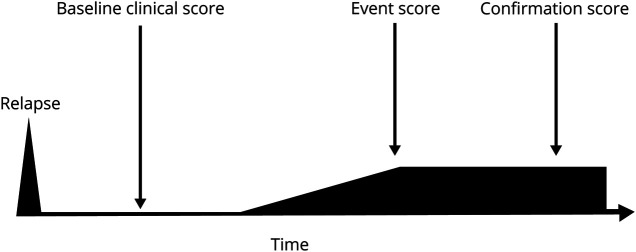
Schematic Representation of PIRA PIRA is the increase in disability of the event score (most commonly EDSS) compared with baseline score. Absence of relapses is required between 90 days before and 30 days after the event score and at the time of the confirmation score. EDSS = Expanded Disability Status Scale; PIRA = progression independent of relapse activity.

The most recent, harmonized definition of PIRA^8^ recommends the following principles for its assessment: (1) an increased disability score (or event score), commonly measured on the Expanded Disability Status Scale (EDSS), should be recorded at least 3 months after and 1 month before the onset of an investigator-reported relapse; (2) a new baseline score should take place after each relapse; and (3) a confirmation score should be assessed at least 3 months after the initial disability increase and 1 month before the onset of an investigator-reported relapse (Figure 2).^8^

This clinical-based definition presents certain challenges. First, the choice of the 3-month interval after relapse onset may not be adequate to “disconnect” the observed disability increase from the preceding relapse. The pathologic correlates of relapses are acute inflammatory demyelinating lesions.^9^ Both Wallerian degeneration of axons following transection in acute lesions, and subsequent loss of chronically demyelinated axons, contribute to postlesional neurodegeneration.^11^ Inflammatory lesions and neurodegeneration are present at all stages of MS.^9^ Although substrates for “primary” neurodegeneration have been proposed,^12^ there is no robust evidence for them. Longitudinal MRI studies have shown ongoing and sustained optic nerve atrophy 1 year after optic neuritis, despite improved visual function.^13^ Optical coherence tomography (OCT) has shown thinning of the retinal fiber layer up to 6 months from acute optic neuritis.^14^ Similarly, spinal cord atrophy occurs over 6 months after acute myelitis, concurrent with clinical recovery.^15^ A sequence of events, unfolding over at least 2 years, arising from white matter lesions to subsequent white matter damage and then gray matter atrophy, has been demonstrated in progressive MS using MRI.^16^ Therefore, it is credible that, at least in some cases, the observed progression designated as PIRA could still be secondary to the preceding relapse, even when measured later than 3 months from relapse onset.

Second, an interval of 1 month before a relapse may not be appropriate to identify progression independent of that relapse. Microstructural MRI changes in the white matter of patients with MS are observed 3–18 months before acute lesions become visible on conventional MRI.^17,18^ These findings suggest that there may be inflammation-related neuronal damage, with consequent neurodegeneration, months before the clinical onset of a subsequent relapse. Therefore, the time window proposed for the definition of PIRA should be interpreted with caution.

Third, the development of asymptomatic brain MS lesions confirms that focal inflammatory demyelination does not always manifest with an acute relapse. Although asymptomatic spinal cord lesions are rarer than asymptomatic brain lesions, they are seen in relapsing MS.^19,20^ All subjects with a radiologically isolated syndrome (RIS) who developed primary progressive MS during a 15-year follow-up possessed spinal cord lesions at the time of the diagnosis of RIS,^21^ suggesting that disability accrual may be induced by concurrent, asymptomatic inflammation, that may not be captured by the current definition of PIRA.^8^

Therefore, on current evidence, it is difficult to justify a disconnect between inflammatory demyelination (relapses) and neurodegeneration (progression). It is possible that some variance in the observed disability accrual that defines PIRA could be linked to the preceding, concurrent, or impending inflammation.

## The Limitations of Incorporating MRI Activity Into PIRA

The terms “true PIRA,” “pure PIRA,” and “PIRMA” (progression independent of relapses and MRI activity) have been proposed to add the absence of brain and spinal cord MRI activity (new T2 lesions and/or gadolinium [Gd] lesions) between 3 months after and 1 month before the onset of a relapse.^4,8^ In a mildly affected cohort of patients with MS, 30.8% of PIRA events were accompanied by MRI activity.^22^ The inclusion of new brain T2 lesions and/or Gd lesions into PIRA has reduced the number of PIRA events by half (from 47.9% to 23.4%),^4^ suggesting that asymptomatic brain lesions may be responsible for at least some PIRA events previously reported. The use of PIRMA is an important step because it aligns the concept of PIRA to the definition of disease activity in terms of both relapses and new brain lesions, commonly adopted in clinical practice and MS research. It also aligns PIRA to no evidence of disease activity, which also incorporates relapses and MRI activity.

However, the inclusion of new brain lesions into the PIRA definition (i.e., PIRMA) also requires deliberation. First, asymptomatic spinal cord lesions occur between 15% and 25% of patients with RRMS without relapses,^19,20^ hence a rigorous PIRMA assessment should include regular spinal cord MRI. Importantly, lesion location may also be crucial, if affecting an eloquent pathway. A single, cord lesion (or >1 lesion), critically situated in the corticospinal tract, may result in asymmetric motor progression in MS with otherwise low lesion burden (<5 lesions).^23^ This suggests that certain CNS regions, such as cord, may be more relevant for progression in MS than other regions or global injury. However, spinal cord MRI is currently not mandatory for the monitoring of MS^24^ and technically challenging for the robust identification of lesions.

Second, the occurrence of new brain lesions on conventional MRI is only one of the inflammatory processes that may contribute to disability accrual in MS; other processes include not only asymptomatic spinal cord lesions^19^ but also chronic active lesions (e.g., paramagnetic rim lesions and slowly expanding lesions^25^) and cortical lesions^26^ (although these appear less inflammatory than white matter lesions).

Third, a clinical relapse is the manifestation of a symptomatic acute lesion, but an asymptomatic new MRI lesion could theoretically produce subclinical worsening, thereby leading us to reconsider the concept of RAW because subclinical disability deterioration after an asymptomatic lesion may be related to that new lesion. This would lead us to reconsider what is meant by the term “relapse” and whether asymptomatic lesions should be included under this term.^27^ The differences between RAW and PIRA may be simply due to the intensity of the inflammatory process.

## The Challenges of Translating PIRA Into Clinical Settings

The use of PIRA remains limited to clinical trials and research (including prospective, longitudinal cohorts) because the determination of PIRA in clinical practice is prohibitively challenging and necessitates regularly scheduled and standardized clinical assessments and comprehensive MRI protocols. Several parameters affect the accuracy characteristics of PIRA.^8^ Robust PIRA assessment requires a frequent number of visits, more than standard clinical care. A confirmation score should be recorded at least 3 months after the event score, but preferably 6 or 12 months.^8^ Relapses should be investigator-reported, rather than patient-reported.^8^ Acute symptoms, which are not commonly recognized as relapses, such as predominant cognitive impairment^28^ and fatigue, can occur, and disability accrual may be linked to these unconventional MS relapses. In addition, the inclusion of all investigator-reported relapses (instead of only EDSS-confirmed or protocol-defined relapses) may lead to fewer PIRA events. In addition, the duration of follow-up affects the number of recorded PIRA events.

There is an aspiration to broaden the definition of PIRA by using a composite outcome and include other clinical manifestations of disability progression, such as cognitive decline, upper and lower limb function and/or deterioration detected by digital tools, and patient-reported outcome measures. These composites will likely increase the sensitivity of PIRA, by encompassing patients who develop progression in domains beyond EDSS, which is highly weighted toward motor disability, but they are likely to further limit its use in everyday practice.

## The Limitations of PIRA as the End Point of Clinical Trials

Several studies have investigated the impact of disease-modifying treatments on PIRA as an outcome.^6,29^ These trials showed that treatment benefit was mostly explained by a reduction in relapse activity and, consequently, RAW, and by reducing development of new MRI lesions, which, in turn, may lower the likelihood of PIRA in the long term. However, in the short-term, treatment trials would not significantly slow down disability progression, possibly due to the effects of existing lesions, and PIRA should continue to be observed despite a reduction in relapse rate. A possible interpretation of this finding is that a treatment suppressing relapse activity, also reduces disability associated with relapses, thereby making it more likely that any observed disability accrual is independent of relapses.^1^ This hypothesis has been confirmed by a recent simulation of a randomized controlled trial (personal communication at ECTRIMS 2023) showing that the placebo arm has fewer PIRA events than the treatment arm because it is more likely that EDSS increases are associated with relapses that are more numerous in the placebo arm than in the treatment arm. Therefore, a treatment with high efficacy on relapses may artificially increase the number of PIRA events compared with placebo,^29^ and accurate detection of all RAW events, made possible with appropriate visit timings, is necessary if PIRA is to be used as a primary end point.

A methodological challenge for PIRA assessment in clinical trials is that baseline EDSS at study entry is likely to reduce over time because of (1) regression to the mean and (2) patients who are enrolled after an acute event may improve over time. A roving baseline, which has been recommended,^8^ cannot be used in randomized clinical trials because a postrandomization event cannot be used as reference value because of possible treatment influence. This consideration suggests that assessing treatment effects on PIRA in randomized clinical trials remains challenging, although not impossible with careful planning and adapted statistical methodology.

## Advanced-PIRMA to Understand Disability Accrual in Multiple Sclerosis

**Figure 3:**
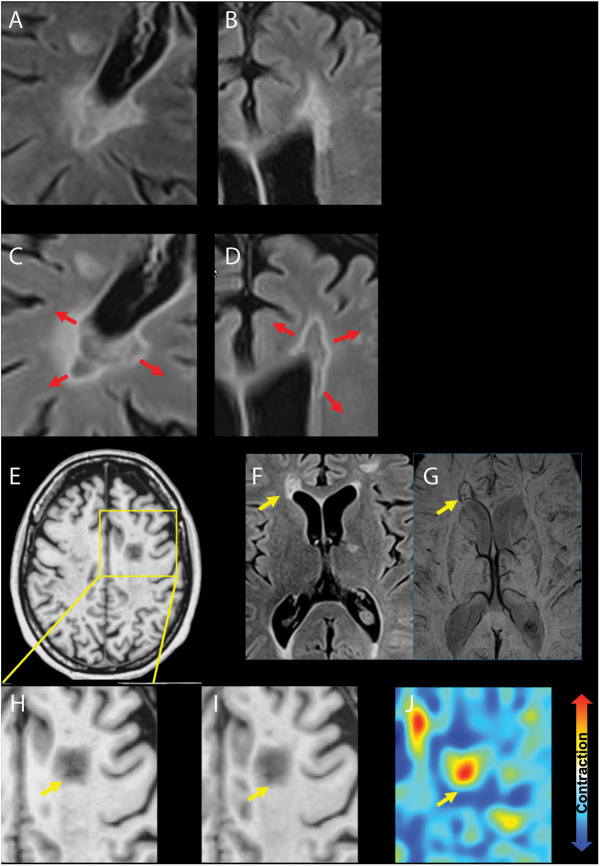
Examples of Slowly Expanding Lesions and a Paramagnetic Rim Lesion Axial FLAIR images showing slowly expanding lesions in the periventricular regions developing between baseline (A and B) and 2-year follow-up (C and D). The red arrows indicate the direction of the lesion expansion. Note also the increase in hypointensity indicating progressive tissue destruction. Axial FLAIR image (F) and SWI (G) showing a paramagnetic rim lesion (yellow arrow). (E) Axial T1-weighted image showing an hypointense lesion in the corona radiata at baseline (H) (yellow arrow) and 2 years follow-up (I) (yellow arrow), with corresponding deformation map (L), which shows in red a concentric expansion of the lesion over time (called slowly expanding lesion) (positive Jacobian >0) and in blue a shrinkage (Jacobian ≤0). FLAIR = fluid-attenuated inversion recovery; SWI = susceptibility-weighted imaging.

**Figure 4:**
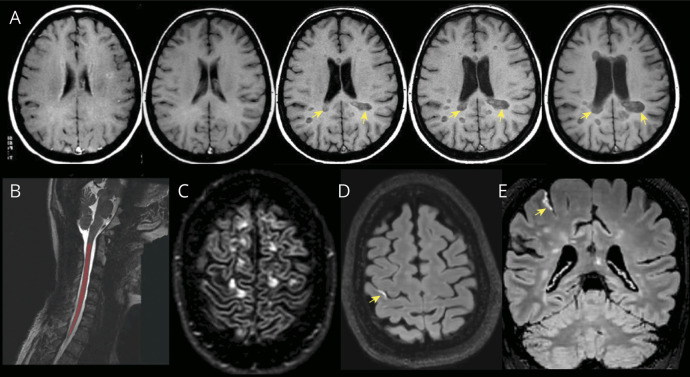
Examples of Brain Atrophy, Slowly Expanding Lesions, Spinal Cord Segmentation, Cortical Lesions, and Leptomeningeal Enhancement (A) Axial T1-weighted images acquired between 1995 (first image on the left) and 2005 (last image on the right) showing progressing development of brain atrophy (increase size of the ventricles and widening cortical sulci) and slowly expanding lesions (yellow arrows). (B) Sagittal T2-weighted image showing one of the steps required for computation of spinal cord longitudinal atrophy, which involves segmentation of the spinal cord (area in red) which is separated from the cerebro-spinal fluid. (C) Axial DIR image showing a couple of intracortical lesions (yellow arrows). (D) Axial and (E) coronal contrast-enhanced 3D T2-FLAIR images showing a high-signal linear hyperintensity (yellow arrow) in the right central sulcus, indicating leptomeningeal enhancement. DIR = double inversion recovery; FLAIR = fluid-attenuated inversion recovery.

The mechanisms of disability accrual not associated with relapses in RRMS and captured by advanced MRI techniques encompass: (1) chronic active lesions, detected on MRI as paramagnetic rim lesions (PRLs),^30^ and slowly expanding lesions (SELs)^31^ (Figures 3 and 4); (2) widespread axonal injury and neuronal loss, reflected by brain atrophy,^32^ cortical and deep gray matter atrophy,^33^ spinal cord atrophy^30^ (Figure 4), and white matter tracts damage on MRI (personal communication at ECTRIMS 2023); (3) cortical lesions^34^ (Figure 4); (4) diffuse and focal microglia activation, revealed by TSPO-PET,^35^ which can also detect chronic active lesions; and (5) leptomeningeal enhancement^36^ (Figure 4), which is nonspecific for MS, but may correspond to meningeal inflammatory infiltrates and microglial activation in the adjacent cortex. The implementation of these advanced imaging techniques in clinical practice is arduous, and the interpretation of these mechanisms can be complicated by other factors, such as compensatory mechanisms related to neuroplasticity or remyelination, and aging. In addition, comorbidities, mainly vascular, are associated with a greater risk of MS progression.^37^

Therefore, we support the use of PIRA to understand the main determinant of disability accrual in large, observational, cohort studies, where regular MRI scans are not included (or are not feasible), and introduce “advanced-PIRMA” to investigate the contributions to disability accumulation of all the underlying pathologic processes, including new brain and spinal cord lesions, PRLs, SELs, brain and spinal cord atrophy, etc., using conventional and advanced imaging, fluid biomarkers, and OCT metrics. Fluid biomarkers, such as serum neurofilament light chain and glial fibrillary acidic protein, whose combined elevation is associated with increased risk of PIRA,^38^ may also play a role in explaining disability accrual. In addition, genetic variants (a genetic determinant of MS severity has recently been discovered^39^) and profiles of specific proteins, such as those related to the arachidonic acid-derived lipid mediators,^40^ are associated with neurodegenerative processes that contribute to disease progression. A corollary of advanced-PIRMA is that detectable residual disability accrual, unexplained after considering all these underlying mechanisms, could highlight crucial unmet areas of future research in MS pathobiology.

## Conclusion and Future Directions

PIRA has been informative in alerting clinicians to the notion that progressive disability accrual occurs across the full spectrum of MS, similar to previous MRI findings of development of brain atrophy in the early stages of MS. However, the interpretation of PIRA as a measure specific to disease progression independent of inflammation cannot fully represent the underlying mechanisms of the disease.

The inclusion of new brain lesions in the PIRA framework (i.e., PIRMA) is an important step that aligns it with other clinical end points but does not address certain fundamental shortcomings: (1) disability worsening may continue for more than 3 months after a relapse and (2) inflammatory processes other than brain T2/Gd lesions (i.e., asymptomatic spinal cord lesions, chronic active lesions) or those not detected by MRI may contribute to the accumulation of disability in MS.

A more accurate assessment of clinical deterioration, beyond EDSS (e.g., composite outcomes or digital biomarkers), which includes cognitive decline and patient-reported outcome measures, is necessary. However, this will make PIRMA assessment even more complex. More consideration should be given to the use of PIRMA as an end point in clinical trials and the consequences for their design and statistical analysis.

Advanced-PIRMA can be applied to understanding the contributions of pathogenic processes studied with imaging (e.g., new brain and spinal cord lesions, SELs, PRLs, intracortical lesions, total brain, spinal cord and gray matter atrophy, leptomeningeal enhancement, diffuse microglia activation), fluid biomarkers, OCT, genetic variants, and proteomics toward disability accrual. Any extant disability progression unexplained by these processes could help focus future research directions.

Our improved understanding of mechanisms of progression and the known relationship between inflammation and neurodegeneration highlight the need to move from a clinical-based definition of PIRA toward a more biologically focused framework,^10^ which roots disability accrual in MS into the underlying pathologic processes (chronic inflammation and neurodegeneration), reflected in vivo by MRI. Despite the challenges posed by the implementation of this proposal in clinical practice, it aligns well with the recent recommendation of characterizing MS based on disease-driving pathogenic mechanisms, rather than the traditional clinical descriptors.^10^ As our understanding of the pathogenesis of MS is refined, it is possible that the term PIRA will need to be completely replaced in the future. A rigorous understanding of the (MRI-based) determinants of PIRA may also help develop more targeted therapies and understand their mechanisms of action. Finally, postmortem findings in a longitudinal cohort of patients enrolled in PIRA-related studies would be important to clarify the mechanisms of PIRA and provide insights into the pathogenesis of MS.

## Appendix Authors

**Table:**

Name	Location	Contribution
Olga Ciccarelli, MD, PhD, FRCP	Queen Square MS Centre, Department of Neuroinflammation, UCL Queen Square Institute of Neurology, Faculty of Brain Sciences, University College London; National Institute for Health and Care Research (NIHR) University College London Hospitals (UCLH) Biomedical Research Centre, United Kingdom	Drafting/revision of the manuscript for content, including medical writing for content; study concept or design
Frederik Barkhof, MD, PhD, FRCR	Queen Square MS Centre, Department of Neuroinflammation, UCL Queen Square Institute of Neurology, Faculty of Brain Sciences, University College London; Centre for Medical Image Computing, University College London, United Kingdom; Department of Radiology and Nuclear Medicine, Amsterdam UMC, Vrije Universiteit Amsterdam, the Netherlands	Drafting/revision of the manuscript for content, including medical writing for content
Massimiliano Calabrese, MD	Department of Neurosciences, Biomedicine and Movement Sciences, University of Verona, Italy	Drafting/revision of the manuscript for content, including medical writing for content
Nicola De Stefano, MD, PhD	Department of Medicine, Surgery and Neuroscience, University of Siena, Italy	Drafting/revision of the manuscript for content, including medical writing for content
Arman Eshaghi, PhD	Queen Square MS Centre, Department of Neuroinflammation, UCL Queen Square Institute of Neurology, Faculty of Brain Sciences, University College London, United Kingdom	Drafting/revision of the manuscript for content, including medical writing for content
Massimo Filippi, MD, PhD	Neuroimaging Research Unit, Division of Neuroscience, and Neurology Unit, Neurorehabilitation Unit, Neurophysiology Service, IRCCS San Raffaele Scientific Institute; Vita-Salute San Raffaele University, Milan, Italy	Drafting/revision of the manuscript for content, including medical writing for content
Claudio Gasperini, MD, PhD	Department of Neuroscience, San Camillo Hospital, Rome, Italy	Drafting/revision of the manuscript for content, including medical writing for content
Cristina Granziera, MD, PhD	Translational Imaging in Neurology (ThINK) Basel, Department of Biomedical Engineering, Faculty of Medicine, University Hospital Basel and University of Basel; Research Center for Clinical Neuroimmunology and Neuroscience Basel (RC2NB); University Hospital Basel and University of Basel, Switzerland	Drafting/revision of the manuscript for content, including medical writing for content
Ludwig Kappos, MD, PhD	Translational Imaging in Neurology (ThINK) Basel, Department of Biomedical Engineering, Faculty of Medicine, University Hospital Basel and University of Basel; Research Center for Clinical Neuroimmunology and Neuroscience Basel (RC2NB); University Hospital Basel and University of Basel, Switzerland	Drafting/revision of the manuscript for content, including medical writing for content
Maria A. Rocca, MD, PhD	Neuroimaging Research Unit, Division of Neuroscience, and Neurology Unit, Neurorehabilitation Unit, Neurophysiology Service, IRCCS San Raffaele Scientific Institute; Vita-Salute San Raffaele University, Milan, Italy	Drafting/revision of the manuscript for content, including medical writing for content
Àlex Rovira, MD, PhD	Section of Neuroradiology, Department of Radiology, Hospital Universitari Vall d'Hebron, Universitat Autònoma de Barcelona, Spain	Drafting/revision of the manuscript for content, including medical writing for content
Jaume Sastre-Garriga, MD, PhD	Multiple Sclerosis Centre of Catalonia, Department of Neurology, Hospital Universitari Vall d'Hebron, Universitat Autònoma de Barcelona, Spain	Drafting/revision of the manuscript for content, including medical writing for content
Maria Pia Sormani, PhD	Department of Health Sciences, University of Genova; IRCCS Ospedale Policlinico San Martino, Genova, Italy	Drafting/revision of the manuscript for content, including medical writing for content
Carmen Tur, MD, PhD	Multiple Sclerosis Centre of Catalonia, Department of Neurology, Hospital Universitari Vall d'Hebron, Universitat Autònoma de Barcelona, Spain	Drafting/revision of the manuscript for content, including medical writing for content
Ahmed T. Toosy, MD, PhD, FRCP	Queen Square MS Centre, Department of Neuroinflammation, UCL Queen Square Institute of Neurology, Faculty of Brain Sciences, University College London, United Kingdom	Drafting/revision of the manuscript for content, including medical writing for content; study concept or design

## Glossary

EDSS: Expanded Disability Status Scale
Gd: gadolinium
MAGNIMS: Magnetic Resonance Imaging in MS
MS: multiple sclerosis
OCT: optical coherence tomography
PIRA: progression independent of relapse activity
PIRMA: progression independent of relapse and MRI activity
PRL: paramagnetic rim lesion
RAW: relapse-associated worsening
RIS: radiologically isolated syndrome
RRMS: relapsing remitting MS
SEL: slowly expanding lesion
